# Transcriptome Sequencing Identifies Novel Immune Response Genes Highly Related to the Severity of Human Adenovirus Type 55 Infection

**DOI:** 10.3389/fmicb.2019.00130

**Published:** 2019-02-06

**Authors:** Wen Xu, Zhe Xu, Lei Huang, En-Qiang Qin, Jie-li Zhang, Peng Zhao, Bo Tu, Lei Shi, Wen-Gang Li, Wei-Wei Chen

**Affiliations:** ^1^Treatment and Research Center for Infectious Diseases, 302 Military Hospital of China, Beijing, China; ^2^Radiation Oncology Center, 302 Military Hospital of China, Beijing, China

**Keywords:** human adenovirus type 55, immune response, transcriptome sequencing, interleukin-1 family, tumor necrosis factor superfamily

## Abstract

Human adenovirus type 55 (HAdV-55) is considered a highly virulent pathogen causing severe and even deadly pneumonia in immunocompetent people. The mechanisms of HAdV-55-induced initiation and progression of severe pneumonia remain ambiguous. In the current study, we endeavored to identify novel immune response genes which are substantially involved in the pathogenesis of severe inflammation in HAdV-55-infected patients. HAdV-55-infected patients with upper respiratory tract symptoms (minor patients) and pneumonia (severe patients) were enrolled. Through transcriptome sequencing and quantitative real-time PCR, the peripheral blood mononuclear cells of the patients were analyzed. We found that the expression of eight genes, including *Il18*, *Il36b*, *Il17rc*, *Tnfsf10*, *Tnfsf11*, *Tnfsf14*, *Tnfsf15*, and *Il1a*, were closely correlated with the severity of HAdV-55 infection. Most of these genes belong to interleukin-1 family or tumor necrosis factor (TNF) superfamily, respectively. The changes in gene expression were confirmed by Western blot assay. Our data will be crucial for deepening the understanding of the pathogenic mechanisms of severe pneumonia in HAdV-55 infection.

## Introduction

Adenoviruses are a class of double-stranded DNA viruses infecting the respiratory tract, eyes, intestines, urinary tract, and nervous system (Khanal et al., 2018). HAdV type 55 (HAdV-55) causes severe pneumonia in immunocompetent people. HAdV-55 has a recombinant genome from HAdV-B11 which is a renal pathogen with the antigenic epitope, and HAdV-B14 which is a respiratory pathogen conferring the cell tropism, biological and pathological properties (Walsh et al., 2010). From 2004 to 2006, three major outbreaks of HAdV-55 infection took place in Turkey, Singapore, and China mainland, eliciting nearly 1000 patients and 2 deaths (Sun et al., 2014). Other recent outbreaks are also reported (Girouard et al., 2011; Zhao et al., 2014).

Since the discovery of HAdV-55, several laboratory investigations have addressed the viral biology such as its genome and clinical manifestations after infection. Persistent high fever, cough and rapid progression of dyspnea were typically reported in the patients infected by HAdV-55 (Tan et al., 2016). However, the mechanisms of the initiation and progression of severe pneumonia remain ambiguous. It has been reported that serum cytokines such as IL-4 and IL-15 were significantly elevated in patients with severe infection (Chen et al., 2014). Another study demonstrates that patients with severe HAdV-55 infections showed significantly higher levels of blood IL-17^+^ CD4^+^ T lymphocytes and higher levels of serum IFN-γ, IFN-α2, IL-4, and IL-10 (Chen et al., 2014). These data suggest that excessive immune reactions or inflammatory responses might induce severe tissue damage in HAdV-55-infected patients. It has been well known that HAdVs stimulate a robust innate immune response, resulting from a wide array of viral effects on cellular pathways (Hartman et al., 2007; Cook and Radke, 2017). Indeed, the viral genome triggers the formation of NLRP3 inflammasome via the stimulation of Toll-like receptor signaling, causing the activation of pro-inflammatory defense mechanism (Barlan et al., 2011a,b; Stein et al., 2012). Thus, a thorough understanding of the inflammatory responses in HAdV-55 infection is important for developing efficacious therapies.

In recent years, transcriptome sequencing has become increasingly popular in new gene/mutation discovery and gene expression profiling. With this technology, a massive change in regulation of liver transcripts with robust up-regulation of immune-related genes was found in HAdV-5 infection in a rodent model (Ying et al., 2015). However, to our knowledge, no special elaboration was conducted to discover inflammation-related genes in HAdV-55 infection using a transcriptome analysis. Therefore, our study aimed to identify novel candidate genes which are substantially involved in the pathogenesis of severe inflammation in HAdV-55-infected patients. To achieve this goal, the transcriptome of peripheral blood mononuclear cells (PBMCs) in HAdV-55-infected patients was sequenced, *de novo* assembled, and used to compare the transcriptome profiles of patients with healthy volunteers. We found that the expression of eight genes, including *Il18*, *Il36b*, *Il17rc*, *Tnfsf10*, *Tnfsf11*, *Tnfsf14*, *Tnfsf15*, and *Il1a*, were closely correlated with the severity of HAdV-55 infection. Most of these genes belong to interleukin-1 family or tumor necrosis factor (TNF) superfamily, respectively. The changes in the expression of TNFSF10, 11, 14, and 15 were further confirmed by Western blot assay. Our data will deepen the comprehension of the pathogenic mechanisms of severe pneumonia post HAdV-55 infection.

## Materials and Methods

### Patient Samples

Seventeen newly diagnosed patients with HAdV-55 infection, including six with pneumonia (group name “Severe”), six with upper-respiratory tract infection symptoms but without pneumonia (group name “Minor”), and five without any symptoms but having high anti-HAdV-55 IgM (group name “Silent”), were recruited from two outbreaks of HAdV-55 infection in 2011–2012 at two military camps. Informed consent was acquired from all patients. The experimental protocols were approved by the ethical committee of 302 Military Hospital of China (license number 2011033D) and conducted in compliance with corresponding regulations and guidelines. Peripheral venous blood was collected from these patients into EDTA-coated collection tubes. The blood mononuclear cells were isolated through the density gradient enrichment using Ficoll-Paque (GE Healthcare) according to the vendor’s manual. The PBMCs were stored at -80°C before further analysis. Blood samples from six age-matched volunteers (health control, HC) were processed in the same way.

### RNA Purification and cDNA Library Preparation

Total RNA was purified from PBMCs using the phenol/chloroform method (Toni et al., 2018). RNase-free DNase I was used to remove residual genomic DNA contamination. RNA purity was checked using the kaiaoK5500^®^ Spectrophotometer (Kaiao, Beijing, China). RNA integrity and concentration were assessed with the RNA Nano 6000 Assay Kit of the Bioanalyzer 2100 system (Agilent Technologies, CA, United States). Only the samples with RNA quantity >2 μg, RNA concentration >200 ng/μl, RNA integrity value >7 were used for transcriptome sequencing. Two microliters RNA per sample was used for the cDNA sample preparations using NEBNext^®^ Ultra^TM^ RNA Library Prep Kit for Illumina^®^ (#E7530L, NEB, United States) following the manufacturer’s recommendations and index codes were added to attribute sequences to each sample. Briefly, mRNA was purified from total RNA using poly-T oligo-attached magnetic beads. Fragmentation was carried out using divalent cations under elevated temperature in NEBNext First-strand Synthesis Reaction Buffer (5×). First strand cDNA was synthesized using random hexamer primer and RNase H. Second strand cDNA synthesis was subsequently performed using the buffer, dNTPs, DNA polymerase I and RNase H. The library fragments were purified with QiaQuick PCR kits and eluted with EB buffer. PCR was then performed to establish the library.

### Library Examination

Insert size was assessed using the Agilent Bioanalyzer 2100 system (Agilent Technologies, CA, United States), and qualified insert size was accurately quantified using StepOnePlus^TM^ Real-Time PCR System (Library valid concentration >10 nM).

### Library Clustering and Data Filtering

The clustering of the index-coded samples was performed on a cBot cluster generation system using HiSeq PE Cluster Kit v4-cBot-HS (Illumina) according to the manufacturer’s instructions. Reads containing the sequence of adaptors (Adapter Polluted Reads), high content of unknown bases (Ns reads) and low-quality reads were removed from the raw data before further analysis to minimize data noise. In brief, the quality of the transcriptome libraries was first evaluated with FastQC v0.11.5. After that, the reads were assessed by standard quality control criteria, and the following reads were removed: (a) reads which were aligned to primers or adaptors, (b) reads with over 15% of low-quality bases (Q ≤19) in one read, (c) reads with over 5% unknown bases (N bases). After filtering, the remaining clean reads were stored in FASTQ format for further mapping (Table 1).

**Table 1:** Sequencing data for each sample.

Samples	Silent	Minor	HC	Severe
Raw reads number	54,366,912	66,856,104	53,894,280	55,572,122
Adapter polluted reads number	1,532,952	2,163,132	5,176,046	1,198,878
Ns reads number	8,880	10,162	2,734	8,252
Low-quality reads number	12,617,778	24,195,916	2,526,008	13,489,608
Clean reads number	40,207,302	40,486,894	46,189,490	40,875,384
Clean reads rate (%)^∗^	73.96	60.56	85.7	73.55


*^∗^Clean data rate (%), clean reads number/raw reads number. This is a representative table of two independent experiments.*

### Data Mapping and Gene Expression Analysis

The clean reads of each group were aligned to the Human Genome version 19 (downloaded from ENSEMBL database^1^) using the software TopHat 2.0.12 plus Bowtie2 with the default parameter settings. Stringent quality control including sequencing data saturation analysis and reads distribution was performed for each sample to ensure the qualification of the sequencing data. Gene expression level was quantified via calculating Fragments per Kilobase per Millon Mapped Fragments (FPKM) as described in previous studies (Solaimani Kartalaei et al., 2015; Fondi et al., 2017; Morimoto et al., 2018). The differential gene expression analysis was conducted using DESeq 1.18.0. The difference in gene expression between two groups was considered significant when the Log2 ratio ≥1 and *q*-value <0.05. The details of gene mapping and expression analysis are introduced in Supplementary Methods.

### Gene Annotation

To comprehensively investigate the potential roles of indicated differentially expressed genes (DEGs), GO enrichment analysis of DEGs were conducted by Hypergeometric test. The details of *GO Enrichment Analysis of DEGs* are depicted in Supplementary Methods.

### Reverse Transcription and Quantitative Polymerase Chain Reaction (RT-qPCR)

The RNA extraction was performed as described above. Reverse transcription was conducted using All-In-One RT MasterMix (Applied Biological Materials Inc.). The following primers were used for quantifying corresponding gene expression: *Tnfsf14*: 5′-acaccactgcactccaacct-3′ and 5′-tgtcccccaagatctgtttc-3′; *Tnfsf11*: 5′-cccaacggtacacgactca-3′ and 5′-cgctagatgacaccctctcc-3′; *Il18*: 5′-tcaccagaggtcaggtgttc-3′ and 5′-ggctcaccacaacctctacc-3′; *Il36b*: 5′-ttttcctagcctcctcacca-3′and 5′-atttccactcaggacccaca-3′; *Tnfsf15*: 5′-gcagacggagataagccaag-3′ and 5′-gactctgggatcagcaggaa-3′; *Il-1a*: 5′-attcaccctggagcacaatc-3′ and 5′-aggggctagatttggagagg-3′; *Il-17rc*: 5′-agctgactcaggggtggag-3′ and 5′-agcccacagactgaccaaac-3′; *Tnfsf10*: 5′-gctgcctggctgacttaca-3′and 5′-aagcaatgccacttttggag-3′. The reaction was performed using Power SYBR^®^ Green Master Mix (Thermo Fisher Scientific) on a CFX384 Touch^TM^ Real-Time PCR Detection System (Bio-Rad, CA, United States) under the following condition: pre-denaturation at 95°C for 10 min, 40 cycles of denaturation at 95°C for 15 s and annealing at 63°C for 30 s. The expression of each gene was normalized to β-actin expression and calculated using the 2^-ΔΔCt^ method.

### Immunoblotting

The proteins were isolated from PBMCs using the Total Protein Isolation Kit-Blood (ITSI-Biosciences, Johnstown, PA, United States) following the vendor’s manual. Protein quantification was conducted using Quick Start^TM^ Bradford protein assay kit (Bio-Rad, CA, United States). A total of 60 μg of proteins from each sample were loaded onto 12% SDS-PAGE gels for electrophoresis. After that, the proteins were transferred onto PVDF membranes in Tris-Glycine transfer buffer at 100 volts at 4°C for 2 h, followed by blocking the membranes for 1 h with TBS-T buffer containing 5% fat-free milk. After three TBST-T washes, the membranes were incubated at 4°C overnight with 5 μg/ml of each of the following antibodies (all from Abcam, Cambridge, United Kingdom): anti-TRAIL (1:2000, Abcam ab42121), anti-RANKL (1:3000, Abcam ab9957), anti-TNFSF14 (1:1000, Abcam ab201094), anti-VEGI (1:1000, Abcam ab64986), and anti-GAPDH (1:2000, Abcam ab8245). The membranes were then washed with TBS-T and incubated with goat anti-mouse IgG or goat anti-rabbit IgG (1:5000. Both from Thermo Fisher Scientific, MA, United States) for 1 h at room temperature. After three washes with TBS-T, the membranes were developed using SuperSignal^®^ West Dura Extended Duration Substrate (Thermo Fisher Scientific, MA, United States), and the chemiluminescence was recorded and scanned using the ChemiDoc XRS+ system (Bio-Rad, CA, United States).

### Statistics

Data were presented as mean ± standard deviation. Unpaired *t*-test or one-way ANOVA was conducted for comparison of mean values among groups. Each experiment was independently repeated three or four times. *N* = 4 to 6 per group. The difference with a *p*-value <0.05 was considered statistically significant.

## Results

### RNA Expression Profiles in HAdV-55-Infected Patients

We carried out whole transcriptome analysis on mRNAs to study the changes in the cell transcriptome profiles of patients with different severity of HAdV-55-infection. One mRNA sample was randomly selected from each group for this analysis. The transcriptomes from patients and healthy donors were sequenced. The low-quality reads, Ns reads and adapter polluted reads were discarded and clean reads were used for mapping (Table 1). After mapping with TopHat 2.0.12 plus Bowtie2, the unmapped reads and multi-mapped reads were excluded, while the uniquely mapped reads were used for expression analysis.

**FIGURE 1:**
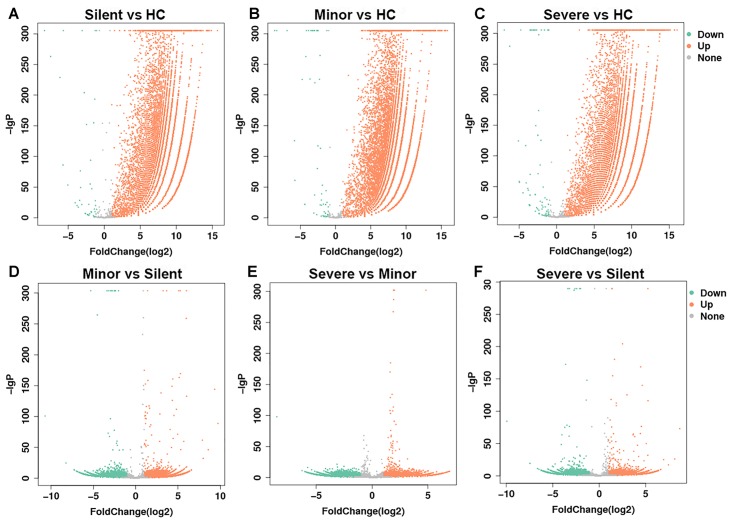
Volcano plot of gene expression changes. The transcriptomes of PBMCs from HAdV-55-infected patients and healthy controls were compared to each other (**A**–**F**, the comparison pairs were noted as the caption of every single volcano plot). The *x*-axis indicates the fold-changes. The *y*-axis displays the negative logarithm to the base 300 of the *t*-test *p*-values. Orange and green dots represent probe sets for transcripts expressed at significantly higher or lower levels in each comparison. HC: healthy control. Silent: patient without any symptoms. Minor: patient with respiratory tract infection symptoms but without pneumonia. Severe: patient with pneumonia.

Before the comparison between patients and healthy donors, mRNAs from two different healthy donors were sequenced and the result suggested that donor-donor variation was not significant (data not shown). In the following gene expression analysis, we mainly focused on the genes of which the expression was up-regulated. As shown in Table 2 and Figure 1, in comparison with the healthy donor, 26,235 mRNAs were upregulated in the “Silent” patient (Figure 1A), 30,235 mRNAs were upregulated in the “Minor” patient (Figure 1B), and 24,831 mRNAs were upregulated in the “Severe” patient (Figure 1C). Only a few mRNAs were down-regulated in each patient. Moreover, as compared with “Silent” patient, 11,766 mRNAs were up-regulated in the “Minor” patient (Figure 1D), while 7247 mRNAs were up-regulated in the “Severe” patient (Figure 1F). The “Severe” patient had 5910 up-regulated mRNAs as compared with the “Minor” patient (Figure 1E). Our data suggest that HAdV-55-infection induced remarkable changes in gene expression in patients’ PBMCs, and the severity of infection also influenced the PBMC gene expression.

**Table 2:** Differentially expressed genes in different comparisons.

	Silent vs. HC	Minor vs. HC	Severe vs. HC	Minor vs. Silent	Severe vs. Silent	Severe vs. Minor
**Up**	26,235	30,235	24,831	11,766	7,247	5,910
**Down**	37	55	76	7,328	7,974	9,986
**Total**	26,272	30,290	24,907	19,094	15,221	15,896


*Up, up-regulation; Down, down-regulation.*

### GO Enrichment of Up-Regulated Genes in Infection

**FIGURE 2:**
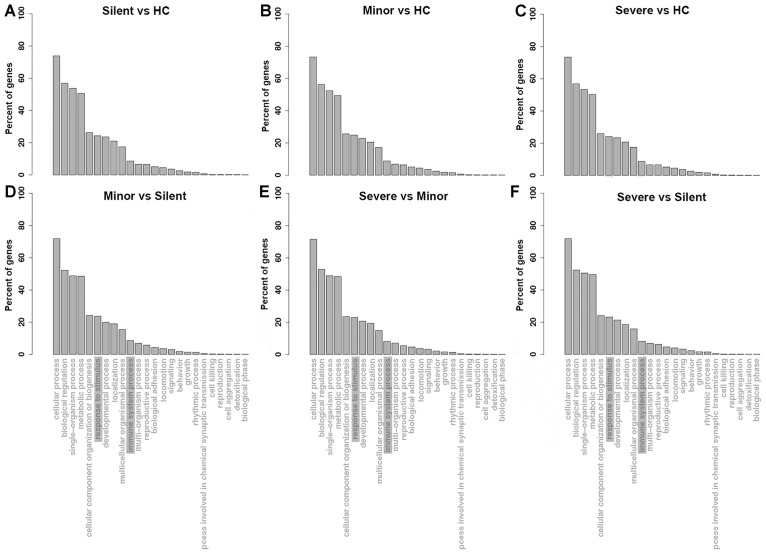
GO enrichment of the up-regulated genes in each comparison (**(A–F)**, the comparison pairs were noted as the caption of every single plot). HC: healthy control. Silent: patient without any symptoms. Minor: patient with respiratory tract infection symptoms but without pneumonia. Severe: patient with pneumonia.

To understand the functions of the up-regulated genes in patients, GO analysis of the up-regulated mRNAs in patients was performed. As shown in Figure 2A–C, with regard to the mRNAs that were upregulated in the “Silent,” “Minor,” and “Severe” patients relative to the healthy control, the GO enrichment showed highly similar patterns. The up-regulated mRNAs were mainly enriched in the biological process of “biological regulation,” “single-organism process,” and “metabolic process.” In addition, several genes were enriched in inflammation-related process, such as “response to stimulus” and “immune system process.” Notably, “response to stimulus” and “immune system process” were also enriched in the biological process in the DEGs of the “Minor” vs. “Silent” comparison (Figure 2D), “Severe” vs. “Minor” comparison (Figure 2E), as well as “Severe” vs. “Silent” comparison (Figure 2F). Therefore, several inflammation-related genes were significantly up-regulated not only in HAdV-55-infection, but also were associated with the severity of the infection.

### RT-qPCR Validation

**FIGURE 3:**
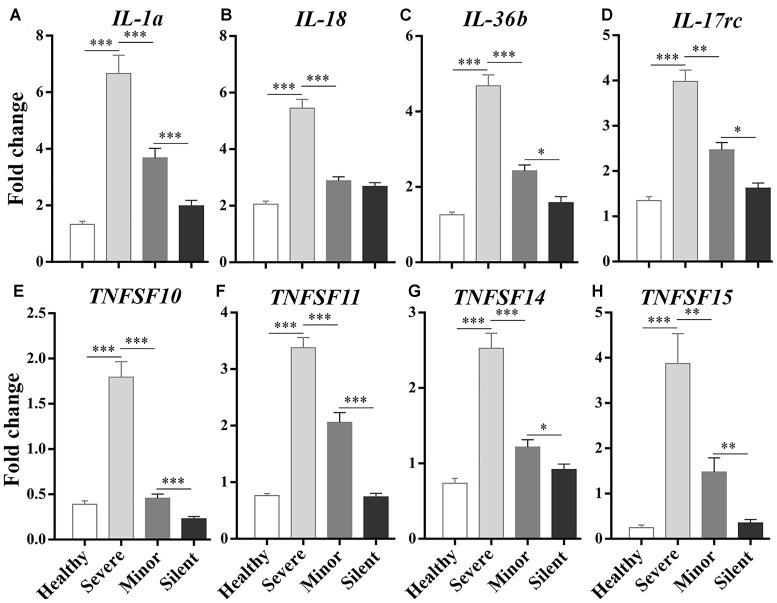
RT-qPCR analysis on the correlations between the expression of inflammatory/immune response genes and the severity of infection. Healthy: healthy control. Silent: patients without any symptoms. Minor: patients with respiratory tract infection symptoms but without pneumonia. Severe: patients with pneumonia. *N* = 5∼6 per group. ^∗^*p* < 0.05; ^∗∗^*p* < 0.01; ^∗∗∗^*p* < 0.001.

To identify the genes that were involved in the inflammatory response after HAdV-55 infection, we focused on the DEGs enriched in “response to stimulus” and “immune system process” in each comparison and checked their fold changes. The up-regulated DEGs with a Log2 ratio ≥1 were first selected in each comparison, and the DEGs encoding inflammatory cytokines or cytokine-associated signaling molecules were further identified from the selected genes. Finally, we figured out several DEGs that were commonly up-regulated in all comparisons, and selected eight most up-regulated DEGs for further investigation, including *Il1a*, *Il18*, *Il36b*, *IL17rc*, *Tnfsf10*, *Tnfsf11*, *Tnfsf14*, and *Tnfsf15* (Supplementary Table 1). To validate the transcriptome sequencing results, these DEGs were analyzed by RT-qPCR. Particularly, the relationship between the expression levels of these genes and the severity of infection was given special attention. We found that their expression levels were positively correlated with the severity of infection, with the highest expression in the “Severe” group (Figure 3). These genes can be roughly divided into two families – interleukin-1 family and TNF superfamily. *Il1a*, *Il18*, and *Il36b* belong to the former gene family. *Tnfsf10*, *Tnfsf11*, *Tnfsf14*, and *Tnfsf15* are the members of the TNF superfamily.

### Immunoblotting Validation

**FIGURE 4:**
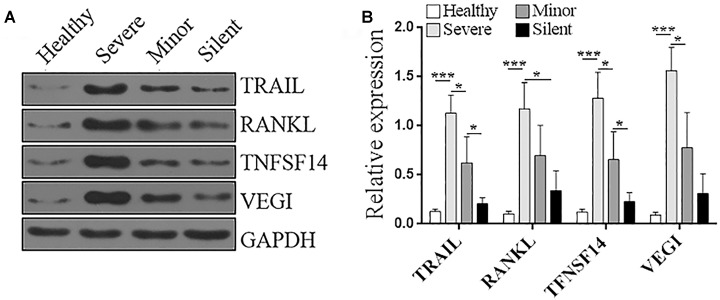
Protein levels of TRAIL, RANKL, TNFSF14, and VEGI in the PBMCs of patients and healthy donors. **(A)** Representative Immunoblotting images. **(B)** Statistics for the protein level of each molecule. The relative expression of each protein was normalized to the GAPDH expression. Healthy: healthy control. Silent: patients without any symptoms. Minor: patients with respiratory tract infection symptoms but without pneumonia. Severe: patients with pneumonia. *N* = 5 per group. ^∗^*p* < 0.05; ^∗∗∗^*p* < 0.001.

Among the above genes, we were interested in the ones that were not reported in adenovirus infection in previous publications. Hence, *Tnfsf10*, *Tnfsf11*, *Tnfsf14*, and *Tnfsf15*, which, respectively, encode TNF-related apoptosis-inducing ligand (TRAIL), receptor activator of nuclear factor kappa-B ligand (RANKL), TNFSF14 (CD258), vascular endothelial growth inhibitor (VEGI), were chosen for the further validation via Immunoblotting. As shown in Figure 4, in the PBMCs of healthy individuals, the protein levels of these molecules were very low. HAdV-55 infection substantially increased the expression of these genes, and their expression levels were positively correlated with the severity of infection, with the highest expression in the “Severe” group. Hence, the Immunoblotting data were consistent with the RT-qPCR results, suggesting that the expression of *Tnfsf10*, *Tnfsf11*, *Tnfsf14*, and *Tnfsf15* indeed reflect the severity of HAdV-55 infection.

## Discussion

Human adenovirus is a double-stranded DNA virus with a diameter of 70∼90 nm (Luiz et al., 2010; Alonso-Padilla et al., 2016). Although intensive studies had shown the epidemic or clinical properties, the immunological aspect of HAdV infection is rarely discussed. Among multiple HAdV genotypes which belong to seven species, HAdV-55 is a pathogen arising from gene recombination between HAdV-11 and HAdV-14 (Walsh et al., 2010; Zhang et al., 2012). According to previous reports, HAdV-55 was more virulent and cause fetal infection. By now, the immunological or inflammatory mechanism of HAdV-55 infection has not been established, and the specific and effective therapies are not available.

Human adenovirus type 55 infection induces complex immune responses, as demonstrated by significantly higher levels of blood IL-17^+^CD4^+^ T lymphocytes and higher levels of serum IFN-γ, IFN-α2, IL-4, and IL-10 (Chen et al., 2014). The IL-17^+^ CD4^+^ T lymphocytes, also known as Th17 cells, play an essential role in inflammatory responses and autoimmunity (Burkett et al., 2015; Patel and Kuchroo, 2015). However, the relationship between the immune responses and pneumonia remains ambiguous. Whether the immune responses, especially the inflammatory reaction, have a profound impact on the severity of HAdV-55 infection is still unclear.

In the current study we analyzed the mRNA profiles of blood leukocytes from HAdV-55-infected patients with distinct infection severity. Interestingly, in comparison with the health control, only a relatively small amount of mRNAs were down-regulated in patients. This might be because that adenovirus-induced innate and adaptive immune responses trigger the activation of a broad spectrum of immune cells including macrophages, granulocytes, dendritic cells, T lymphocytes, and B lymphocytes (Chirmule et al., 1999; Cotter et al., 2005; Hendrickx et al., 2014; Atasheva and Shayakhmetov, 2016). The activated immune cells profoundly promote the expression of genes related to proliferation, microbicidal activity and the inflammatory responses. Therefore, perhaps during the reaction of HAdV infection, the primary reaction of immune cells is to express more immunity-or-inflammation-associated proteins, while a tiny fraction of proteins related to immune tolerance or anti-inflammation are transcriptionally down-regulated. This is also why we focused on the up-regulated mRNAs, since these mRNAs reflect the active immune response or inflammation. Through a comprehensive transcriptome sequencing, we identified eight genes of which the expression was significantly up-regulated and positively associated with the infection severity. *Il18*, *Il36b*, *IL17rc*, *Tnfsf10*, *Tnfsf11*, *Tnfsf14*, *Tnfsf15*, and *Il1a* were the up-regulated genes we discovered. Among them, *Il36b* and *Tnfsf15* are newly identified inflammation-related genes in HAdV-55 infection. Our results suggest new gene markers and therapeutic targets for HAdV-55-induced pneumonia.

*Il1a* encodes IL-1α, a cytokine that has already been implicated in the response to adenovirus infection. In response to HAdV-5, macrophage secret IL-1α to trigger IL-1RI-dependent production of pro-inflammatory cytokines and chemokines (Di Paolo et al., 2009). The arginine-glycine-aspartic acid (RGD) motifs of HAdV-5 interact with macrophage β_3_ integrins to elicit the IL-1α-mediated response (Di Paolo et al., 2009; Fitzgerald, 2009). In addition, IL-1α can facilitate CXCR2-mediated recruitment of neutrophils into the splenic marginal zone during HAdV-5 infection (Di Paolo et al., 2014). Thus, IL-1α is a key factor responsible for the activation of pro-inflammatory responses to adenovirus. Interestingly, blockade of IL-1 signaling can improve the toxicity profile of Ad5-based vectors Ad5L and Ad5/35L (Shayakhmetov et al., 2005), suggesting that IL-1α might be associated with the severity of adenovirus infection. Therefore, our data seem consistent with previous research showing that IL-1α is positively correlated with the extent of adenovirus-induced inflammation. However, IL-1α could also be beneficial for the host immune reaction against viral infection, because IL-1R-deficient mice demonstrated an impaired immune response to vaccinia virus infection (Tian et al., 2017). In our study, we found that *Il1a* mRNA was positively related to the severity of HAdV-55 infection, suggesting that HAdV-55 triggers macrophages to produce IL-1α. Further investigations are needed to elucidate the role of IL-1α in HAdV-55-induced inflammation especially pneumonia.

*Il-18* is a member of the interleukin-1 family. It encodes IL-18 which contributes to the pathogenesis of autoimmune diseases and inflammation. Previous studies suggest that IL-18 is mainly secreted by monocytes/macrophages in reaction to viral or bacterial stimuli (Bossu et al., 2000; Boraschi and Dinarello, 2006). After stimulation, monocytes/macrophages form inflammasomes in their cytosol and process immature IL-1β and IL-18 into their active mature forms (van de Veerdonk et al., 2011). Mature IL-1β and IL-18 are then secreted out of monocytes/macrophages and bind to their receptors on target cells to activate NF-κB and induce inflammatory mediators such as IFN-γ, adhesion molecules, chemokines and Fas ligand (Kaplanski, 2018). In this manner, IL-18 is involved in the activation of Th1, Th2, NK cells, IL-17-producing γδ T cells, and macrophages. Our data show that *Il-18* mRNA was highly expressed in severe HAdV-55 infection. It is therefore very likely that HAdV-55 infection induces the transcription of IL-18 in monocytes/macrophages through toll-like receptor (TLR)-related signaling and inflammasome formation, and the expression of IL-18 represents the extent of anti-viral reaction and is closely correlated with the infection severity.

*Il36b* encodes IL-36β (also known as interleukin-1 family member 8), which is another member of the interleukin-1 family. IL-36β is considered to be expressed by monocytes/macrophages, T lymphocytes and B lymphocytes. Nevertheless, the stimuli which induce IL-36β expression remain unclear. It has been reported that other members of IL-36 cytokines such as IL-36α and IL-36γ are secreted by keratinocytes, monocytes, or epithelial cells after stimulation with epidermal growth factor, TLR agonists or inflammatory stimuli (Barksby et al., 2009; Bochkov et al., 2010; Franzke et al., 2012; Gabay and Towne, 2015). It is likely that IL-36β is produced in a stimulus-dependent manner in inflammatory responses. However, the regulatory mechanisms of the IL-36β expression in different disorders remain to be elucidated. We found that *Il36b* mRNA was closely related to the severity of HAdV-55 infection. To our knowledge, we are the first to report the up-regulation of IL-36β expression in HAdV-55 infection. The target cells of IL-36β are dendritic cells and CD4+ T lymphocytes (Franzke et al., 2012). It has been reported that IL-36 family members upregulate CD80, CD86, and MHCII and induce the production of IL-12, IL-1β, IL-6, TNF-α, and IL-23 in dendritic cells. Furthermore, IL-36 family members enhance CD4+ T cells to produce IFN-γ, IL-4, and IL-17a (Vigne et al., 2011). Since IL-36α, IL-36β, and IL-36γ bind to the same receptor complex, it is reasonable to speculate that IL-36β is also involved in the activation of innate and adaptive immune responses in HAdV-55 infection. The exact role of IL-36β in the host defense against HAdV-55 invasion remains unidentified and would be elucidated in further investigations.

*Tnfsf10*, *Tnfsf11*, *Tnfsf14*, and *Tnfsf15* encode TRAIL, RANKL, TNFSF14 (CD258), and VEGI, respectively. TRAIL is a crucial protein secreted by various tissue cells including cells of the immune system such as natural killer (NK) cells, T cells, natural killer T cells (NKT cells), dendritic cells and macrophages (Falschlehner et al., 2009). It induces apoptosis of target cells via binding to death receptor 4 and death receptor 5. It has been reported that TRAIL expression is increased in these cells upon activation with different stimuli (Fanger et al., 1999; Kayagaki et al., 1999; Wang et al., 2001; Ehrlich et al., 2003). Hence, the TRAIL expression level likely reflects the extent of both innate and adaptive immune response, and severe viral infection could elicit robust immune responses and subsequently a high serum TRAIL level. Our findings are consistent with this hypothesis, demonstrating that *Tnfsf10* mRNA was robustly expressed in the PBMCs of patients with severe HAdV-55 infection. Indeed, it has been indicated that TRAIL can act as a biomarker for distinguishing between bacterial and viral infections, since it is consistently up-regulated in virus-infected patients (Oved et al., 2015). However, how HAdV-55 infection up-regulates TRAIL expression has not been determined yet. It is also unknown whether HAdV-55 influence TRAIL-mediated apoptosis of virus-infected tissue cells. Previous studies reveal that Ad5-E1A gene product sensitizes cells to TRAIL-dependent killing, whereas E3 gene products, and to a lesser extent E1B-19K, inhibit this effect (Routes et al., 2000). Whether HAdV-55 induces similar effects needs to be studied in future.

RANKL is widely expressed in distinct tissues and cells including leukocytes. In particular, it is expressed by T helper cells and it mediates dendritic cell survival and maturation. B cells are another primary source of RANKL in certain conditions (Kawai et al., 2006; Titanji, 2017). The role of RANKL in anti-viral immunity has been demonstrated in previous research. RANKL is critical for the induction of anti-viral CD8+ effector T cells (CTL) during cutaneous herpes simplex virus infection (Finsterbusch and Piguet, 2015). RANKL/RANK signaling axis in the protective immune responses in the spleen marginal zone is important for the host response to viral infection and induction of acquired immunity (Habbeddine et al., 2017). However, the role of RANKL/RANK signaling in adenovirus infection has not been well studied. Since we found that *Tnfsf11* mRNA was significantly higher in “severe” and “minor” patients, we speculate that in HAdV-55 infection, RANKL is up-regulated in reactive T cells, B cells and macrophages, and the RANKL expression level is positively associated with the infection severity.

Interestingly, TNFSF14 was also closely related to the severity of HAdV-55 infection in our investigation. It is well known that TNFSF14 is expressed on various cell types such as T cells, dendritic cells, macrophages, and NK cells, and LIGHT-induced signaling leads to NF-κB-driven gene activation and inflammatory reactions (Sedy et al., 2014). Hence, TNFSF14 is a cytokine crucial for the initiation and progression of inflammation. Indeed, increased serum levels of light/tnfsf14 in non-alcoholic fatty liver disease suggests that TNFSF14 potentially promoting hepatic inflammation (Otterdal et al., 2015). Moreover, the herpes virus entry mediator, which is the receptor of TNFSF14, is linked to dysregulated mucosal function such as pneumonia (Shui and Kronenberg, 2013). Given that severe HAdV-55 infection also causes pneumonia, it is likely that TNFSF14 participates in the HAdV-55-induced inflammation in lungs.

To our surprise, VEGI was found to be positively associated with the severity of HAdV-55 infection. VEGI is a cytokine predominantly expressed in endothelial cells and lamina propria (LP) macrophages but not T cells and B cells. It has been paid attention in the tumor study because it inhibits angiogenesis which is critical for tumor growth (Parr et al., 2006; Zhang et al., 2009, 2010). It is later known that VEGI plays an active role in the modulation of immunity and inflammation. VEGI stimulates T cell activation, Th1 cytokine production, and dendritic cell maturation through death receptor 3 (Zhang and Li, 2012). Additionally, VEGI-mediated signaling favors Th2 cytokine production in the lung, and act as a critical trigger for allergic lung inflammation (Fang et al., 2008). Moreover, VEGI also promotes Th17 cell functions (Pappu et al., 2008). Hence, the increase in VEGI expression in HAdV-55-infected patients probably contributes to the anti-viral response and favors elimination of virus-containing target cells, but might aggravates pneumonia as well. Interestingly, VEGI expression can be stimulated by TNF-α and IL-1 (Chew et al., 2002; Migone et al., 2002; Kang et al., 2005). So it is highly possible that up-regulation of VEGI is a secondary change caused by the initial inflammation after HAdV-55 infection.

Moreover, IL-17RC was also shown to be up-regulated in severe and minor HAdV-55 infection. IL-17RC is a member of the IL-17 receptor family. Other members of this family include IL-17RA, IL-17RB, IL-17RD, and IL-17RE. IL-17RC pairs with IL-17RA to form a heterodimeric receptor which recognizes IL-17A and IL-17F (Jin and Dong, 2013). The IL-17 family cytokines are active players in inflammatory responses and autoimmune reactions (Jin and Dong, 2013). Although the significance of IL-17RC in HAdV-55-induced pneumonia is unidentified, recent studies indicated that the lung epithelium express IL-17RC which promotes respiratory allergy or pulmonary defense against *Klebsiella pneumoniae* (Chen et al., 2016; De Luca et al., 2017). The up-regulation of IL-17RC on the lung epithelium might enhance the inflammatory response in the lung after HAdV-55 invasion, and therefore reflects the extent of pneumonia.

In a recent research, a bioinformatics analysis revealed the close phylogenetic relationship among all HAdV-55 strains. Interestingly, although HAdV-55 strains have high genome identity, the genetic variation such as single-nucleotide non-synonymous substitutions, synonymous substitutions, insertions, and deletion in non-coding regions occurs between different HAdV-55 isolates. In particular, the major non-synonymous substitutions occurs in the protein pVI, which has different functions at various stages of an adenovirus infection (Cheng et al., 2018). Viral genetic variation is caused by multiple mechanisms such as genetic recombination, gene duplications, gene exchanges and gene adoptions. The most common variation is nucleotide substitution as a result of polymerase error in reading the template in the replication process (Sanjuan and Domingo-Calap, 2016). Interestingly, no non-synonymous substitutions are present in the major capsid proteins of different HAdV-55 strains (Cheng et al., 2018), suggesting that the genetic variation might not influence the host immune response to HAdV-55 strains.

Collectively, our research demonstrates the genes that are highly correlated with the severity of HAdV-55 infection. Further studies focusing on those genes will help understand the inflammation mechanism of HAdV-55 infection. Our data might shed light on the pathogenesis of HAdV-triggered pneumonia, and provide new clues for the diagnosis and therapy of HAdV infection.

## Author Contributions

W-GL and W-WC conceived the study, participated in its design and coordination, and drafted the manuscript. WX carried out the molecular biological analysis and the manuscript writing, participated the design of the study and statistical analysis. ZX, LH, and E-QQ carried out the cases collection and participated the design of the study. J-lZ, PZ, BT, and LS participated in the clinical data acquisition and specimen collection. All authors read and approved the final manuscript.

## Conflict of Interest Statement

The authors declare that the research was conducted in the absence of any commercial or financial relationships that could be construed as a potential conflict of interest.

## Footnotes

1 http://www.ensembl.org/index.html
