# Supplementary material for: Transcriptome Sequencing Identifies Novel Immune Response Genes Highly Related to the Severity of Human Adenovirus Type 55 Infection
Source: Front Microbiol. 2019 Feb 6;10:130. doi: 10.3389/fmicb.2019.00130 (PMC6372566; doi:10.3389/fmicb.2019.00130)
Supplement: Supplementary file 1 [file Table_1.DOCX]

Supplementary Material

Transcriptome sequencing identifies novel immune response genes highly related to the severity of human adenovirus type 55 infection

Wen Xu^1^, Zhe Xu^1^, Lei Huang^1^, En-Qiang Qin^1^, Jie-li Zhang^1^, Peng Zhao^1^, Bo Tu^1^, Lei Shi^1^, Wen-Gang Li^2^^*^, Wei-Wei Chen^1*^

^1^ Treatment and Research Center for Infectious Diseases, 302 Military Hospital of China, Beijing, China, 100039

^2^ Radiation Oncology Center, 302 Military Hospital of China, Beijing, China, 100039

**Corresponding authors:**

Wei-Wei Chen

Treatment and Research Center for Infectious Diseases, 302 Military Hospital of China, Beijing, China, 100039

Email: cww302@126.com

Phone: +86 4006-111-30240

Wen-Gang Li

Radiation Oncology Center, 302 Military Hospital of China, Beijing, China, 10003906-111-302

Email: doctor302@163.com

Phone: +86 4006-111-302

**Supplementary methods**

# Quality Control

Raw Data was processed with Perl scripts to ensure the quality of data used in further analysis. The adopted filtering criteria are as follows˖

1. Remove the adaptor-polluted reads (Reads containing more than 5 adapter-polluted bases were regarded as adaptor-polluted reads and would be filtered out).
2. Remove the low-quality reads. Reads with the number of low quality bases (phred Quality value less than 19) accounting for more than 15% of total bases are regarded as low-quality reads;
3. Remove reads with number of N bases accounting for more than 5 %.

As for paired-end sequencing data, both reads would be filtered out if any read of the paired-end reads are adaptor-polluted.

The obtained Clean Data after filtering will be carried out on statistics analyses on its quantity and quality, including Q30, data quantity and base content statistics, etc.

# Sequence Alignment

The reference genome and the annotation file were downloaded from ENSEMBL database [(http://www](http://www.ensembl.org/index.html)).[ensembl.org/index.html).](http://www.ensembl.org/index.html)) Bowtie/Bowtie2 was used for building the genome index, and Clean Data was mapped to the reference genome using TopHat v2.0.12. TopHat, specialized software for transcriptome sequencing reads mapping, can identify exon-exon junctions by splitting the mapped reads and mapping them to the reference genome again. Moreover, TopHat calls Bowtie/Bowtie2 for mapping, which makes it more accurate and fast.

# Mapping File Visualization

The IGV (Integrative Genomics Viewer) was used to view the mapping result by the Heatmap, histogram, scatter plot or other style.

# Quantitation of Gene Expression Levels

Fragments Count for each gene in each sample was counted by HTSeq v0.6.0, and FPKM (Fragments Per Kilobase Per Million Mapped Fragments) was then calculated to estimate the expression level of genes in each sample, the formula is shown as


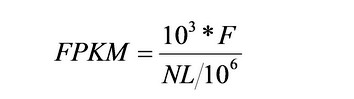


F is the number of fragments in a certain sample that is assigned to a certain gene, N is the total number of mapped fragments in the certain sample and L is the length of the certain gene. FPKM could eliminate the effect of sequencing depth and gene length on gene expression levels and the data could be compared between each other directly.

# Differential Gene Expression Analysis

DEGseq v1.18.0 was used for differential gene expression analysis between two samples with non-biological replicates. Under the assumption that the number of reads derived from a gene (or transcript isoform) follows abinomial distribution, DEGseq is proposed based on MA-plot and widely used for differential gene expression analysis. The P-value could be assigned to each gene and adjusted by the Benjamini and Hochberg’s approach for controlling the false discovery rate. Genes with q≤0.05 and |log2_ratio|≥1 are identified as differentially expressed genes (DEGs). DESeq/DESeq2 was used for differential gene expression analysis between two samples with biological replicates using a model based on the negative binomial distribution. The P-value could be assigned to each gene and adjusted by the Benjamini and Hochberg’s approach for controlling the false discovery rate. Genes with q≤0.05 and |log2_ratio|≥1 are identified as differentially expressed genes (DEGs).

# GO and KEGG Enrichment Analysis of DEGs

# The GO (Gene Ontology, <http://geneontology.org/)>enrichment of DEGs was implemented by the hypergeometric test, in which p-value is calculated and adjusted as q-value, and data background is genes in the whole genome. GO terms with q<0.05 were considered to be significantly enriched. GO enrichment analysis could exhibits the biological functions of the DEGs.

# Supplementary Tables

| **KO number^1^** | **Score^2^** | **Gene** | **Protein** |
| --- | --- | --- | --- |
| K05477 | 6048.937929 | *Tnfsf14* | LIGHT; tumor necrosis factor ligand superfamily member 14 |
| K05473 | 4891.804029 | *Tnfsf11* | RANKL; tumor necrosis factor ligand superfamily member 11 |
| K05482 | 3785.928384 | *Il18* | interleukin 18 |
| K05486 | 2574.185908 | *Il36b* | interleukin 1 family, member 8 (eta) |
| K05478 | 1922.109109 | *Tnfsf15* | VEGI; tumor necrosis factor ligand superfamily member 15 |
| K04383 | 1727.376938 | *Il1a* | interleukin 1 alpha |
| K05166 | 1679.093488 | *Il17rc* | IL17RL; interleukin 17 receptor C |
| K04721 | 1578.977843 | *Tnfsf10* | TRAIL; tumor necrosis factor ligand superfamily member 10 |
| K05168 | 1568.040637 | *Il17re* | interleukin 17 receptor E |
| K04722 | 1565.414658 | *Tnfrsf10* | tumor necrosis factor receptor superfamily member 10 |
| K05487 | 1271.448423 | *Il1f9* | IL1RP2; interleukin 1 family, member 9 |
| K05480 | 772.374617 | *Eda* | ectodysplasin-A |
| K05470 | 707.2113083 | *Tnfsf7* | CD70; tumor necrosis Factor Ligand Superfamily Member 7 |
| K05145 | 704.0724907 | *Nfrsf8* | CD30; tumor necrosis factor receptor superfamily member 8 |
| K05155 | 622.3698388 | *Tnfrsf19* | TROY; tumor necrosis factor receptor superfamily member 19 |
| K04519 | 585.9609745 | *Il1b* | interleukin 1 beta |
| K02583 | 546.097202 | *Ngfr* | nerve growth factor receptor (TNFR superfamily member 16) |
| K04722 | 368.2502875 | *Tnfrsf10* | TRAILR; tumor necrosis factor receptor superfamily member 10 |
| K05446 | 356.8994877 | *Il26* | interleukin 26 |
| K05444 | 335.1390975 | *Ltb* | TNFC; lymphotoxin beta (TNF superfamily, member 3) |
| K05484 | 277.9439482 | *Il1f6* | interleukin 1 family, member 6 (epsilon) |
| K04389 | 205.8924284 | *Tnfsf6* | FASL; tumor necrosis factor ligand superfamily member 6 |
| K03160 | 194.0553782 | *Tnfrsf5* | CD40; tumor necrosis factor receptor superfamily member 5 |
| K05476 | 88.89557723 | *Tnfsf13b* | TNFSF20; tumor necrosis factor ligand superfamily member 13B |
| K05469 | 12.98100392 | *Tnfsf4* | OX40L; tumor necrosis factor ligand superfamily member 4 |

# Supplementary Table 1. Commonly up-regulated DEGs in all comparisons

1. KO number: the KEGG Orthology number assigned to each protein.
2. The score is the sum of the fold change values obtained by comparing the KEGG: KO signal pathways associated with the pfam:pfam_Name genes.
